# Unit Costs in Health Economic Evaluations: Quo Vadis, Austria?

**DOI:** 10.3390/ijerph20010117

**Published:** 2022-12-22

**Authors:** Susanne Mayer, Agata Łaszewska, Judit Simon

**Affiliations:** 1 Department of Health Economics, Center for Public Health, Medical University of Vienna, Kinderspitalgasse 15/1, 1090 Vienna, Austria; 2 Department of Psychiatry, University of Oxford, Warneford Hospital, Oxford OX3 7JX, UK

**Keywords:** health care costs, societal perspective, unit costs, patient and family costs, productivity loss, database, Austria

## Abstract

Evidence-informed healthcare decision-making relies on high quality data inputs, including robust unit costs, which in many countries are not readily available. The objective of the Department of Health Economics’ Unit Cost Online Database, developed based on systematic reviews of Austrian costing studies, is to make conducting economic evaluations from healthcare and societal perspectives more feasible with publicly available unit cost information in Austria. This article aims to describe trends in unit cost data sources and reporting using this comprehensive database as a case study to encourage relevant national and international methodological discussions. Database analysis and synthesis included publication/study characteristics and costing reporting details in line with the Consolidated Health Economic Evaluation Reporting Standards (CHEERS 2022) with the year of the database launch as the cut-off point to assess how the methods have developed over time. Forty-two full economic evaluations and 278 unit costs were analyzed (2004–2016: 34 studies/232 unit costs, 2017–2022: 8 studies/46 unit costs). Although the reporting quality of costing details including the study perspective, unit cost sources and years has improved since 2017, the unit cost estimates and sources remained heterogeneous in Austria. While methodologically standardized national-level unit costs would be the gold standard, a systematically collated list of unit costs is a first step towards supporting health economic evaluations nationally.

## 1. Introduction

For evidence-informed healthcare decision-making, high-quality data inputs, such as reliable unit cost estimates, are necessary. A few countries have therefore developed national-level unit cost programs with publicly available lists of unit cost estimates, including the UK [1] and the Netherlands [2]. These countries typically have a longer tradition in the systematic application of health economic evaluations, and the unit cost databases are in high demand. For instance, the unit costs for health and social care estimated and collated by the Personal Social Services Research Unit (PSSRU) at the University of Kent are referenced in 68% of UK economic evaluations [3]. In the Netherlands, using the reference price costs developed by the National Health Care Institute is mandatory for all reimbursement dossiers [2]. 

In Austria, the absence of a nationwide unit cost program substantially curbs the feasibility and comparability of health economic evaluations [4]. Although Austrian policy makers advocate for the use of evidence-based health technology assessment (HTA) [5], the available evidence base is not yet at the needed level and continues to lag behind international trends [6,7]. As a first step in 2016, the Department of Health Economics (DHE; Center for Public Health) at the Medical University of Vienna therefore developed a Microsoft Excel-based publicly accessible, comprehensive catalogue of unit costs, their sources and other relevant meta-information as reported in published Austrian economic evaluations and costing studies (DHE Unit Cost Online Database, 2004–2015) [4]. The data were retrieved from existing studies based on a systematic review of the peer-reviewed and grey national literature to draw a complete picture of the Austrian health economics and unit cost landscape. The database has been regularly updated since. The goal of the DHE Unit Cost Online Database is to help lower the barrier to conducting economic evaluations from healthcare and societal perspectives by raising awareness of existing costing sources, their limitations and costing reporting standards. 

The database can be accessed for free [8], and between its launch (02/2016) and the end of the current observation period (09/2022) it has been downloaded 370 times by members of research institutions (n = 226, 61%), for-profit organizations (n = 41, 11%), governmental or other national public institutions (n = 37, 10%), health care providers (n = 22, 6%), and others (n = 44, 12%), averaging approximately 50 downloads annually. While 69% of the users are from Austria, 31% report being from an international primary institutional affiliation. Thus, there appears to be a clear demand for readily available Austrian unit cost estimates.

A similar unit cost database collection drawing on existing sources, but based on a different identification strategy and broader in scope by covering additional sectors including ‘education and skills’, ‘crime’ and ‘energy’, is available for the UK [9]. On an international scale, the European Healthcare and Social Cost Database (EU HCSCD) collates data for a core set of health and social care cost items from existing sources for nine European countries (England, France, Germany, Italy, Poland, Portugal, Slovenia, Spain, Sweden) [10]. Likewise, lists of selected unit costs by country and/or disease area are available in the peer-reviewed literature (e.g., [11,12]). For instance, in Germany no official unit cost program exists, but several standardized cost lists were published [13,14].

Some of the methodological issues related to costing in Austria have been highlighted in earlier studies. For example, a costing exercise based on the unit cost of a general practitioner (GP) consultation demonstrated that different costing approaches and sources may be justified depending on the adopted study perspective [15]. Yet, this aspect is hardly reflected in existing economic evaluations according to a previous systematic literature review in 2015 [4]. In that review, it was also pointed out that the relevant costing details and unit costs themselves are often not adequately reported according to the Consolidated Health Economic Evaluation Reporting Standards (CHEERS) [4]. Another study confirmed that the main challenges in Austrian economic evaluations are related to methodological aspects including data quality [6]. These findings, however, are not unique to Austria but have been established in similar international reviews [11,16,17,18,19,20,21,22]. What remains unclear from these reviews, however, is if the situation has improved in recent years—especially in the context of the introduction of unit cost lists—and what the next steps could be to further enhance the quality of unit cost data for use in economic evaluations.

This article aims to describe the state of the art and present trends in Austrian economic evaluations regarding unit cost data and their reporting standards based on the CHEERS (2022) checklist analyzing the information included in the comprehensive DHE Unit Cost Online Database. The database’s initial publication year was selected as the cut-off point to analyze how the costing methods have developed over time (2004–2016 versus 2017–2022), and it was hypothesized that the unit cost reporting quality has improved since the introduction of the database. Such an assessment will help determine the status quo and identify and raise awareness of methodological problem areas in Austria and beyond. Internationally, this case study could stimulate the discussion about necessary future steps to further increase the quality of economic evaluations regarding unit costing, both in countries with and without existing unit cost libraries.

## 2. Materials and Methods

For the first version of the DHE Unit Cost Online Database (v1.1) launched in February 2016, a comprehensive systematic literature review of the Austrian peer-reviewed literature was conducted and published [4]. Multiple bibliometric databases (MEDLINE, EMBASE, the Social Science Citation Index, EconLit, Scopus) and grey literature from key national HTA stakeholders were searched. The Guideline for Conducting Systematic Literature Reviews in Economic Evaluation [23] and the Preferred Reporting Items for Systematic reviews and Meta-Analyses (PRISMA) statement [24] were followed. All economic evaluations of healthcare interventions in Austria were considered for inclusion.

Out of the 93 studies identified in the previous systematic review [4], 71 were deemed suitable for the DHE Unit Cost Online database and were included. For example, economic evaluations that did not report any unit costs separately from the resource use information (the ‘ingredients approach’) were excluded, as the main purpose of the database is to present unit costs together with relevant metadata. Studies only reporting medication costs and patient-related average costs were also excluded from the database for two reasons: medication costs are readily available from published sources including the official Austrian classified index of goods (‘Warenverzeichnis’ [25]). This source is commonly cited in the Austrian literature [4], albeit costing of prescription and over-the-counter medication may in fact be more complex depending on the costing perspectives [26]. Patient-related average costs over time were excluded, as they are often disease-specific and different from unit costs per service unit, for example, and the aim of the database was to present the latter.

The extracted data collated in the Microsoft Excel (2013) DHE Unit Cost Online Database include the ‘description of unit cost’, ‘unit of measure’, ‘unit cost’, ‘range of unit cost’, ‘year of unit cost’, ‘sector of unit cost/cost category’, ‘regional level of unit cost’, ‘original source(s) of unit cost’, ‘explanation/limitation of unit cost’, ‘study disease’, ‘study perspective’, ‘study type’ and ‘study reference (original study)’ per each resource use item listed in a study. The database is accompanied by an introductory text, a database guide describing the headings, together with a database sample screenshot for illustrative purposes. Any input data included in the database was independently double-extracted by two researchers, and any differences were resolved via joint discussion.

The systematic literature review leading to the DHE Unit Cost Online Database entries in 2016 (2004–2015, v1.1) has been regularly updated since (in 2017 for year 2016, v2.1; in 2019 for 2017–2018, v3.1; in 2022 for 2019–04/2022, v4.1), following the same methodological gold standards as in the original review [4] but with an improved search strategy (applied in MEDLINE, EMBASE, Scopus, Psyndex, and the Social Science Citation Index). The search details for MEDLINE are presented in File S1.

This article synthesizes the full economic evaluations (cost-effectiveness analyses, cost-utility analyses, cost-benefit analyses, cost-minimization analyses, cost-consequence analyses [27]), and their costing data listed in the DHE Unit Cost Online Database (v4.1). The analyzed data cover the publication characteristics, study characteristics and costing reporting details in line with the CHEERS 2022 checklist [28]. CHEERS contains several items relevant for costing, including the statement of the analytical study perspective, transparency about the adopted methodology for the “valuation of resources and costs and their data sources”, and the reporting of the “date […] of unit costs” [28] (p. 19).

To prepare the data in the DHE Unit Cost Online Database for analysis, additional columns were introduced for the purpose of this review, for example ‘disease area per study according to the International Statistical Classification of Diseases and Related Health Problems (ICD, 10th Revision, [29])’, ‘journal impact factor (year of publication or 2021 if published later) according to the Journal Citation Report (JCR)’, ‘study funding’ (public sector, for profit organizations, not stated/no funding), ‘clear reporting of sector of the unit cost’, ‘clear reporting of unit cost source’ and ‘type of unit cost source [4]’. New data entries were double-extracted independently by two authors (S.M., A.Ł.) and discrepancies were discussed collaboratively.

Due to the small number of included studies, any assessment of trends (2004–2016 versus 2017–2022) are based on descriptive information (change in percentages) only. To test for differences in reporting standards on the cost level, chi-square tests of independence between the two periods were carried out in Stata 17.

Although a clear cut-off time point was selected for the analysis, studies published between 2017 and 2022 (henceforth referred to as: after 2017) may still have had their analyses carried out before the database launch due to a potential lag in the publication process [30]. A sensitivity analysis was conducted excluding the studies published in 2017 and 2018 and using unit costs from the period 2004–2016 (henceforth referred to as ‘before 2017’) (n = 3).

## 3. Results

**Figure A1:**
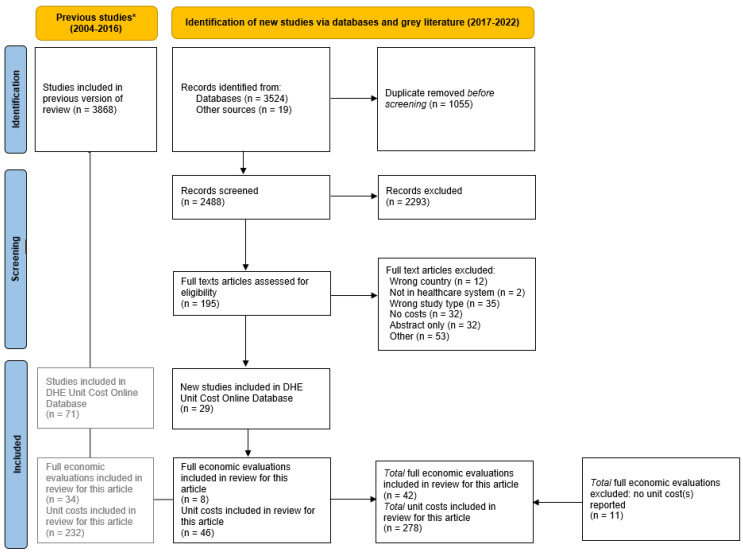
Modified PRISMA flow chart. * Updated information form [4]. Flow chat modified from [51].

A PRISMA flow chart detailing the number of identified, screened and included studies and unit costs is presented in Appendix A (Figure A1). Out of the 100 studies included in the database (v4.1), 42 studies are full economic evaluations and hence are included in this review. Thirty-four studies were published before 2017 and eight studies were published between 2017 and 2022.

Table 1 outlines the main characteristics of the 42 studies. After 2017, more studies (100% vs. 74% before 2017) in the database were journal articles, with more articles (75% vs. 59%) published in JCR-listed journals and in the English language rather than in German (88% vs. 68%). The mean impact factor was higher for the JCR-indexed articles after 2017 (3.03 vs. 2.43).

The study characteristics of the full economic evaluations remained similar over time, with cost-effectiveness and cost-utility analyses being the dominant types in both periods, while relatively more studies were model-based rather than trial-based after 2017 (75% vs. 62%). By disease area, most studies covered the ICD chapters ‘II Neoplasms’ (50% vs. 26%), ‘IX Diseases of the circulatory system’ (13% vs. 15%) and ‘X Diseases of the respiratory system’ (13% vs. 12%). The funding source of the studies was also similar over time, with an equal percentage of studies financed by for-profit organizations (such as the pharma industry) and the public sector (e.g., the Main Association of Austrian Social Security Institutions) before and after 2017, respectively.

In terms of relevant costing information and reporting details in accordance with the CHEERS checklist (Table 2), the proportion of studies lacking information on the adopted analytical perspective decreased from 35% (before 2017) to zero (after 2017). After 2017, more studies stated a payer perspective (100% vs. 59%) and fewer studies were conducted from a societal viewpoint (13% vs. 26% before 2017). In line with this, before 2017, more studies reported unit costs beyond the health and social care sectors, namely from the patient and family domain related to, for example, out-of-pocket expenses (9% vs. 0% after 2017), and lost productivity due to absenteeism (24% vs. 13% after 2017).

A total of 278 unit costs from full economic evaluations are listed in the database (before 2017: 232, after 2017: 46), and are predominantly from the healthcare sector. The five most common health and social cost categories (n = 264) are inpatient costs (37%, n = 97), followed by outpatient (8%, n = 21), general practitioner (8%, n = 20), specialist (6%, n = 17), and outpatient ward contacts (5%, n = 13). This categorization also highlights one of the problems of imprecise or insufficient labelling of the specific health and social care segment that the unit cost refers to. With specialist care being provided both in physician practices or within a hospital setting in Austria [31], the explicit specification of the segment (and specialty) is crucial to avoid ambiguity, especially since the unit cost may differ considerably. For instance, the listed unit costs for an (unspecified) specialist consultation ranged from EUR 20 to 105 in 2021 prices (inflated based on the Austrian Consumer Price index, [32]).

Before 2017, the reported years of unit costs ranged from 2000 to 2014, and after 2017 from 2012 to 2018. The reporting of the year of unit cost improved with all unit cost years after 2017 being clearly stated (vs. 79% before 2017). A similar tendency can be observed regarding the unit cost source(s). After 2017, 63% (vs. 53% before 2017) of the source details were comprehensively and clearly reported and zero sources were unclear (vs. 28% before 2017). Examples of the unclear referencing of sources (before 2017) include vague specifications like “case fee” and “flat fee”, and ambiguous references include “average diagnosis-related group (DRG) tariff” and “hospital’s cost accounting” lacking further details.

After 2017, all valuation sources were payer tariffs (vs. 48% before 2017), including tariff catalogues from regional sickness funds and data provided by the Main Association of Austrian Social Security Institutions for the outpatient sector, and reimbursement tariffs provided by hospital organizations for inpatient services. Before 2017, the studies additionally drew on provider-specific costs (11%) via hospital controlling departments and other sources (7%) including official reports/statistical offices, unit costs in other articles, and market prices. With this, even for the same resource use, reported sources vary across studies, as do unit costs. For example, based on three different sources in three studies, the unit cost for an (unspecified) visit to a hospital outpatient ward ranged from EUR 34 to EUR 100 in 2021 prices [32].

Before 2017, one in five unit costs (18%) were either based on expert opinion/author assumptions or the type of source could not be determined with the given information, compared to zero after 2017. Before 2017, expert opinion and author assumptions were the most common sources for disease-specific procedures and stays in specific hospital wards.

## 4. Discussion

This review painted a mixed picture about the state of the art and unit costing reporting quality of full economic evaluations included in the Austrian DHE Unit Cost Online Database. On the one hand, and in line with the research hypothesis, the presentation of unit cost data has improved in recent years, including the reporting of the relevant CHEERS details such as the study perspective, the year of the unit cost and the exact source. Studies no longer seem to need to draw on assumptions and expert opinions as unit cost sources. On the other hand, the costing sources used even for identical resource use items remain heterogeneous, potentially resulting in considerably differing unit cost estimates between studies, as shown in this review by way of example. As a basic prerequisite for such an assessment, though, unit costs need to be fully reported. This is still not the case in all studies (Figure A1): A total of 11 identified full economic evaluations (in relation to the 42 included full economic evaluations, accounting for 26% of them) lacked relevant unit cost data or did not follow the ‘ingredients approach’. Hence, they could not be included in the DHE Unit Cost Online Database to begin with.

In general, these mixed findings compare well with the international literature. For example, a previous review that aimed to identify unit costs for health economic evaluations of diabetes in France, Germany and Italy found that two-thirds of cost-effectiveness analyses reported unit costs and 40% provided references to all unit costs, while 30% provided references for only some or none of the unit costs [11]. The authors concluded that in the centralized system (e.g., France), nationwide cost data sources were available for almost all resource use items of interest and the unit cost sources were considered good quality, while in a decentralized system (e.g., Italy), multiple sources for each resource use item were available with varying cost data quality [11]. This observation is also valid in our study, and the variety of unit cost sources and heterogeneity of unit cost estimates may reflect the fragmented Austrian health care system. A study from Brazil found that 37% of reviewed economic evaluations reported resource use and unit costs separately [17]. This was also the only study [17] looking at trends over time, suggesting that the reporting quality of economic evaluations in general increased significantly since 1980 [17]. A less favorable picture of the reporting quality was presented in a review of economic evaluations in developing economics, which stated that only 21% of evaluated studies scored “excellent” in conduct and reporting cost estimates [16].

Thus, several practical problem areas concerning unit cost reporting prevail nationally and internationally, and their detailed analysis using Austria as a case study may provide some indication as to what directions the field may be heading in the future (‘quo vadis’), both in Austria and beyond. Note that some of the proposed ways forward have previously been explored in the EU-funded ProgrammE in Costing, resource use measurement and outcome valuation for Use in multi-sectoral National and International health economic evaluAtions (PECUNIA; 2018–2021) [33,34,35], in which all co-authors of this manuscript were involved (PI: J.S.).

Firstly, the clarity of the unit cost reporting regarding the exact source and hence the potential reliability of the unit cost still leaves room for improvement. Specifically, providing a full reference comprehensible for anybody without insider knowledge of the national system for each reported unit cost would also introduce the necessary transparency for an external quality assessment. In contrast, citing, for example, the DHE Unit Cost Online Database as the only reference without providing further details on the original unit cost sources is considered insufficient. As part of this review, four studies were identified that listed the DHE Unit Cost Online Database as the primary source and the original references listed in the database as secondary sources.

Secondly, more awareness is needed regarding the ambiguity of reporting unit costs without an explicit specification as to which healthcare segment the unit cost refers to. A novel solution to do away with such vagueness was recently implemented in PECUNIA by incorporating and extending the DESDE (Description and Evaluation of Services and DirectoriEs) framework [36] for the standardized service description [37]. Generally, applying such a taxonomical coding system allows moving away from the simple naming of resource use, which can be ambiguous [38], and was also shown to be insufficient in this review. For example, using DESDE, a unit cost for a specialist consultation, could be assigned a specific outpatient, day care, or residential code element for clarity. This code would then also enable the international comparability of services based on activities, which is required for a valid likeness of the services captured, for example, in multi-national economic evaluations [34]. The successful implementation of such a coding system in the standard health economics toolbox may, however, be challenging, as establishing a culture of coding for service descriptions as in ecosystems research is still in the early stages [38].

Thirdly, the unit cost sources used in Austrian economic evaluations for comparable services remain heterogeneous. This also holds true in Germany, where publishing cost lists reportedly failed to resolve issues related to the lack of standardization of costing in economic evaluations [39]. At the same time, why different sources for the same unit cost may be justified was recently explored in the context of a GP consultation in Austria [15]. However, using, for example, tariffs as a unit cost source excluding patients’ out-of-pocket expenses does not fully reflect a societal perspective. Currently, such methodological reflection is missing in published studies. As pointed out earlier [15], to avoid unjustified variation in unit cost sources, more methodological and theoretical guidance in this regard seems necessary, for example, in any future update of the (non-mandatory) Austrian pharmacoeconomic guidelines [40] from 2006 and the HTA methods handbook [41] from 2012 [6]. Alternatively, national and international researchers may prefer to resort to methodologically harmonized, societal reference unit costs (RUCs) altogether, as covered in the PECUNIA RUC Compendium [42], available since July 2021. The multi-sectoral, multi-country PECUNIA RUC Compendium contains RUCs for a core set of resource use items in the health, social care, education, (criminal) justice and employment sector as well as the patient, family and informal care domain from six European countries, including Austria [43,44]. These RUCs were also externally validated, which is another advantage of the PECUNIA RUCs over, for example, non-validated unit costs with potentially uncertain reliability. The PECUNIA RUCs, which aim to reflect societal opportunity costs, were developed based on harmonized costing tools, including the PECUNIA RUC templates [45,46,47]. This transparent methodological foundation will allow for low-threshold updates and extensions to further services and countries in the future.

The PECUNIA RUC Compendium might further support the feasibility of economic analyses from a broad multi-sectoral perspective, which have been scarce in recent years according to this review. To conduct economic evaluations from a societal viewpoint, unit costs from various sectors are a prerequisite. However, the DHE Unit Cost Online Database currently lacks estimates for resource use beyond the health and social care sectors for so-called inter-sectoral costs and benefits (ICBs) [48]. For instance, for any study investigating the costs and outcomes of a mental-health intervention on the educational attainment of children or the use of criminal justice services by adults, no unit cost would be readily available in the current version of the Austrian database due to the lack of inclusion in the underlying studies. This limited scope might imply that ICBs are also less likely included in future studies. A recent survey among Dutch researchers revealed a considerable discrepancy in this respect: Although the majority of the respondents claimed that the inclusion of ICBs in economic evaluations is important, only a minority have previously done so [49]. At the same time, the Netherlands is one of the few countries with comprehensive, published unit costs for ICBs and a compulsory societal perspective [50].

### Limitations

The data synthesis presented in this paper has several limitations. Firstly, albeit making for a considerable proportion of studies in the DHE Unit Cost Online Database (52%, n = 37 before 2017 and 72%, n = 21 after 2017), partial economic evaluations were excluded from this review. As the CHEERS checklist does not apply to all forms of economic evaluations covered in the DHE Unit Cost Online Database [27] and the comparability of partial and full economic evaluations in terms of reporting was found challenging in a previous review [4], only full economic evaluations were included to allow for more homogenous study comparison. Secondly, due to the exclusion criteria of the DHE Unit Cost Online Database, economic evaluations reporting medication costs (only), per-patient costs (only) and economic evaluations not reporting any unit costs were a priori excluded from this review. As a result, such a study selection does not necessarily allow for a full assessment of the health economic landscape in Austria (as previously presented in [4]). It may paint a more favorable picture of the status quo of unit costing-related reporting standards, but this should have no bearing on any of the conclusions drawn from the study comparison over time. Finally, a sensitivity analysis accounting for a potential publication lag excluded three studies published in 2017 and 2018. The findings were confirmed.

## 5. Conclusions

Albeit a limited step in terms of scope and quality to lower the barrier for conducting health economic evaluations by supporting studies with regards to costing data needs, the list of published unit costs collated in the DHE Unit Cost Online Database was met with great interest by the Austrian (and international) target community. Some unit costing reporting standards in full economic evaluations have improved since the launch of the database. At the same time, other problem areas prevailed, including lacking clarity of the unit cost source, healthcare segment and, related to this, an inability to check the reliability of unit costs and heterogeneous costing sources for identical services. In terms of ‘quo vadis, Austria’, more methodological guidance in this regard may be required in the future to further improve the quality of economic evaluations. Methodologically standardized reference unit costs may be the gold standard. However, a systematically compiled list of unit costs and assessment and reporting of their limitations can serve as a first step in any jurisdiction without, for example, a national-level unit cost program. Such an initiative could help support the feasibility and quality of health economic evaluations in the future.

## Figures and Tables

**Table 1:** Publication and study characteristics (n = 42).

	2004–2016		2017–2022		Trend
	n	%	n	%	
**Publication type**	**(34)**		**(8)**		
Journal article	25	74	8	100	↗
JCR (Journal Citation Report) indexed	20	59	6	75	↗
Non-JCR indexed	5	15	2	25	↗
Report	9	26	0	0	↘
**Publication language**	**(34)**		**(8)**		
English	23	68	7	88	↗
German	11	32	1	13	↘
**Type of full economic evaluation ^1^**	**(34)**		**(8)**		n. r.
Cost-effectiveness analysis	28	82	5	63	
Cost-utility analysis	7	21	3	38	
Cost-minimization analysis	2	6	0	0	
Cost-consequence analysis	1	3	0	0	
**Model-based studies**	21	62	6	75	↗
**Disease area (ICD-10)**	**(34)**		**(8)**		n. r.
II Neoplasms	9	26	4	50	
IX Diseases of the circulatory system	5	15	1	13	
X Diseases of the respiratory system	4	12	1	13	
V Mental and behavioral disorders	4	12	0	0	
XXI Factors influencing health status and contact with health services	3	9	0	0	
Other ICD-10 chapters	9	26	2	25	
**Study funding source**	**(34)**		**(8)**		n. r.
Public sector	12	38	3	35	
For-profit organizations	12	38	3	35	
Not stated/no funding	10	24	2	30	

Trend: ↗ = percentage value increased, ↘ = percentage value decreased, n r. = trend not reported. ^1^ Multiple types are possible, % values do not add up to 100%.

**Table 2:** Unit costing methods and reporting (n = 42 on study level, n = 278 on unit cost level).

	2004–2016		2017–2022		Trend	*p*-Value ^3^
	n	%	n	%		
**Study perspective (n = 42) ^1^**	**(34)**		**(8)**			-
Payer	20	59	8	100	↗	
Provider	1	3	0	0	↘	
Patient	3	9	0	0	↘	
Societal	9	26	1	13	↘	
Not stated	12	35	0	0	↘	
**Included sectors per study (n = 42) ^2^**	**(34)**		**(8)**			-
Health and social care	34	100	8	100	-	
Patient/family	3	9	0	0	↘	
Productivity losses	8	24	1	13	↘	
**Reporting of unit cost source (n = 278)**	**(232)**		**(46)**			*p* < 0.001
Clear	124	53	29	63	↗	
Reported but exact source unclear/ambiguous	44	19	17	37	↗	
Unclear	64	28	0	0	↘	
**Type of valuation source(s) per unit cost (n = 278)**	**(232)**		**(46)**			*p* < 0.001
Payer tariff	113	49	46	100	↗	
Provider-specific costs	26	11	0	0	↘	
Other sources	16	7	0	0	↘	
Expert opinion/author’s assumption	22	9	0	0	↘	
Unclear type of source	21	9	0	0	↘	
Multiple sources	34	15	0	0	↘	
**Reporting of unit cost year (n = 278)**	**(232)**		**(46)**			*p* < 0.001
Clear	184	79	46	100	↗	
Unclear	48	21	0	0	↘	

^1^ Multiple study perspectives are possible, % values do not add up to 100%. ^2^ Multiple sectors are possible, % values do not add up to 100%. ^3^ Based on chi-square test. ↗ = percentage value increased. ↘ = percentage value decreased.

## Data Availability

Data supporting reported results can be retrieved from: https://healtheconomics.meduniwien.ac.at/downloads/dhe-unit-cost-online-database/ (accessed on 2 June 2022).

## Supplementary Materials

The following supporting information can be downloaded at: https://www.mdpi.com/article/10.3390/ijerph20010117/s1, File S1: Search strategy (MEDLINE).
