# Supplementary material for: Unit Costs in Health Economic Evaluations: Quo Vadis, Austria?
Source: Int J Environ Res Public Health. 2022 Dec 22;20(1):117. doi: 10.3390/ijerph20010117 (PMC9819362; doi:10.3390/ijerph20010117)
Supplement: Supplementary file 1 [file ijerph-20-00117-s001.zip › ijerph-2073734-supplementary.pdf]

Ovid MEDLINE(R)

1 (cost* or economic* or price* or financ*).ti,ab,kw.

2 (health* or medic*).ti,ab,kw.

3 1 and 2

4 (cost* adj3 method').ti,ab,kw.

5 (economic adj3 evaluation*).ti,ab,kw.

6 reimbursement.ti,ab,kw.

7 exp Cost-Benefit Analysis/

8 "cost-benefit analysis".ti,ab,kw.

9 exp "Cost of Illness"/

10 "cost of illness".ti,ab,kw.

11 (cost adj3 control).ti,ab,kw.

12 exp "Costs and Cost Analysis"/

13 "cost-effectiveness analysis".ti,ab,kw.

14 "cost-utility analysis".ti,ab,kw.

15 (cost* adj3 estimate*).ti,ab,kw.

16 (cost* adj3 variable*).ti,ab,kw.

17 exp "models, economic"/

18 3 or 4 or 5 or 6 or 7 or 8 or 9 or 10 or 11 or 12 or 13 or 15 or 16 or 17

19 exp Austria/

20 austria*.ti,ab,kw.

21 (Graz or Innsbruck or Vienna or Wien or Salzburg or Linz or tirol or Tyrol or Styria or Carinthia or "Lower Austria" or "Upper Austria" or Vorarlberg or Burgenland).ti,ab,kw.

22 19 or 20 or 21

23 18 and 22

24 limit 23 to dt=20160101-20161231
